# Microfluidic Concentric Gradient Generator Design for High-Throughput Cell-Based Studies

**DOI:** 10.3389/fbioe.2017.00021

**Published:** 2017-04-12

**Authors:** Elishai Ezra Tsur, Michal Zimerman, Idan Maor, Avner Elrich, Yaakov Nahmias

**Affiliations:** ^1^Grass Center for Bioengineering, The Hebrew University of Jerusalem, Jerusalem, Israel; ^2^Neuro-Biomorphic Engineering Lab, Faculty of Engineering, Jerusalem College of Technology, Jerusalem, Israel; ^3^Department of Cell and Developmental Biology, The Hebrew University of Jerusalem, Jerusalem, Israel

**Keywords:** biomems, computer-aided design, numerical modeling, high-content studies, soft lithography

## Abstract

Gradients of diffusible signaling molecules play important role in various processes, ranging from cell differentiation to toxicological evaluation. Microfluidic technology provides an accurate control of tempospatial conditions. However, current microfluidic platforms are not designed to handle multiple gradients and cell populations simultaneously. Here, we demonstrate a rapidly adaptable microfluidic design able to expose multiple cell populations to an array of chemical gradients. Our design is based on pressure-equilibrated concentric channels and a pressure-dissipating control layer, facilitating the seeding of multiple cell populations in a single device. The design was numerically evaluated and experimentally validated. The device consists of 8 radiating stimuli channels and 12 circular cell culture channels, creating an array of 96 different continuous gradients that can be simultaneously monitored over time.

## Introduction

Gradients of diffusible signaling molecules play important roles in numerous processes, both biological and chemical. For example, it was shown that biochemical gradient signaling such as growth factors and hormones can result in directed cell migration and chemotaxis (Ahmed et al., 2010; Lee et al., 2011). Predominantly, in biological developmental processes, the notion of morphogen gradient is intimately associated with paradigms on the determination of the cell’s location, differentiation, and fate (Cosson and Lutolf, 2015). Chemical gradients are also used extensively in toxicity screening, where the influence of different drug concentrations on cell function is analyzed to derive dose–response curves (Prill et al., 2016). Hence, there is a great interest in platforms that can maintain variable, stable tempospatial gradients (Kim et al., 2010; Somaweera et al., 2016).

Since Whitesides and colleagues proposed in 1999 to utilize microfluidic technology for gradient generation (Takayama et al., 1999), various designs have emerged, showing promising potential as a powerful and versatile technology to accurately control tempospatial conditions (Weibel and Whitesides, 2006). Generally, two design paradigms of microfluidic gradient-generating devices were developed: flow based and diffusion based (Chung and Choo, 2010). Flow-based devices use laminar flow and diffusive mixing to create a chemical gradient. Some platforms are based on sequentially mixing and splitting a solution in a microchannel, resulting in a stable concentration gradient in a single compartment (Somaweera et al., 2016). Other designs are using microfluidic “jets,” injection of minute amounts (pLs) of fluid into an open pool to generate gradients on open surfaces (Keenan et al., 2006). These platforms are limited by their inability to minimize the hemodynamic forces impacting the cells and their incompatibility to create gradient profiles in three-dimensional (3-D) scaffolds.

Diffusion-based devices use dispersal patterns of solutions to create a gradient, allowing molecular diffusion between two continuously replenished channels with chemical solutions and plain medium. Various designs of diffusion-based microfluidic gradient-generating devices were proposed. Keenan et al. (2010) presented a design of an interconnected three-chambered device. Although this design achieves a stable diffusion-based gradient, it can only generate a single gradient in one chamber. Irimia et al. (2006) used a series of parallel dividers in the longitudinal direction of a microfluidic channel to restrict the diffusion between two initial parallel streams of distinct concentrations, creating a chemical gradient in the direction transversal to the channel. Li et al. (2007) demonstrated the generation of linear and non-linear concentration gradients along microfluidic channel using computer-controlled additions of samples to the channel. Atencia et al. (2009) designed a device termed “the microfluidic palette,” which is capable of generating multiple spatial chemical gradients simultaneously inside a single chamber. While those designs allow generation of complex gradient patterns, they are limited to the creation of a pattern in a single chamber. Other designs use rigid membranes and hydrogels to restrict flow while allowing diffusion (Liu et al., 2012). While hydrogels are especially appealing because they support 3-D cellular structures and migration, current designs are limited in throughput and have restricted dynamics of the gradient tempospatial conditions due to slow diffusion time.

Here, we propose an innovative microfluidic design capable of a rapidly generating various chemical gradients in multiple pressure equilibrated channels, each can be seeded with different cell lines. This computer-controlled, pressure automated design consists of 12 circular channels for different cell lines and 8 radiating stimuli channels, achieving an array of 96 gradients that can be monitored in real-time simultaneously and enabling measurements of numerous data points in varying time frames.

## Methods

### Numerical Modeling

A computational fluid dynamic (CFD) model was used to model gradient generation via chemical diffusion within the 3-D model of the device. Geometry was designed using AutoCAD and meshed using 5 µm tetrahedral elements (extremely fine mesh). CFD simulations were carried out by Comsol Multi-physics 4.3b, coupling the stationary Navier–Stokes module for fluid dynamics with the convection and diffusion model for transport of diluted species. Inlet chemical concentration was set to 1 mol/m^3^ with a diffusion coefficient (*D*) of 1.8 × 10^9^ m^2^/s. Fluid density was defined as 10^3^ kg/m^3^, with a dynamic viscosity of 10^3^ Pa s.

### Device Fabrication

The device was fabricated using photolithographically defined molds. First, a 60-µm reagent layer and a 40-µm cell layer were created using reflowed positive photoresist AZ-4652 (Micro-Chem, MA, USA) and a negative photoresist SU-8 (Micro-Chem, MA, USA), respectively. Both patterned on a single silicon wafer. The reflowed reagent layer creates a circular channel, enabling its tight sealing, as was previously described by Melin and Quake (2007). Next, a 40-µm control layer that consists of a single air pressure-controlled microstructured channel complex was molded on a different silicon wafer and fabricated separately. Both molds were fabricated in a class-100 clean room environment (HUJI Center for Nanoscience and Nanotechnology). Control layer was cast by replica molding of PDMS (Sylgard 184; Dow Corning, MI, USA). Flow layer was spin coated with PDMS, allowing precise control of the layer thickness. The control layer was aligned on top of the flow layer, forming a 5- to 6-µm membranes, where the control and flow channels intersect orthogonally, creating a push-down valve. Layers were bonded together via curing agent diffusion. Process was previously described in detail by Melin and Quake (2007).

### Device Control and Imaging

Perfusion was controlled via an integrated microfluidic controller, based on the Microsoft Gadgeteer micro-processor (GHI Electronics, MI, USA), which can set the pressure regulators and configure the outlets of manifolds via its analog and digital outputs. Control system was described in length in Ezra et al. (2015). Pressure regulators and manifolds were purchased from Festo, Israel. Pressurized fluid containers were purchased from Fluigent, UK. Device was imaged using fluorescence LSM700 microscopy (Zeiss, Germany) and perfused with 1:50 diluted Rhodamine (2 × 10^6^ particles/mL) (Sigma-Aldrich, Israel) at velocities ranging from 0.001 to 0.1 m/s. Results were quantified using Zeiss ZEN software.

### Cell Culture

The device was precoated with 860 μg/mL rat tail type I collagen (BD Biosciences, CA, USA) diluted in 0.02 N acetic acid. Caco-2 cells (ATCC, VA, USA) were perfused in a concentration of 14 million cells/mL to the device at a flow rate of 25 µL/min. Cells were incubated in the device with daily media changes for 7 days in a 5% CO_2_-humidified incubator at 37°C. In day 7, viability of cells was quantified using LIVE/DEAD viability assay (Invitrogen Life Sciences, CA, USA), in which the cytoplasm of live cells accumulates green-fluorescent calcein because of esterase activity, while the nuclei of dead cells are labeled red by ethidium homodimer owing to a loss of nuclear membrane integrity. Cells were quantified using ImageJ.

## Results

### Design and Operation

**Figure 1:**
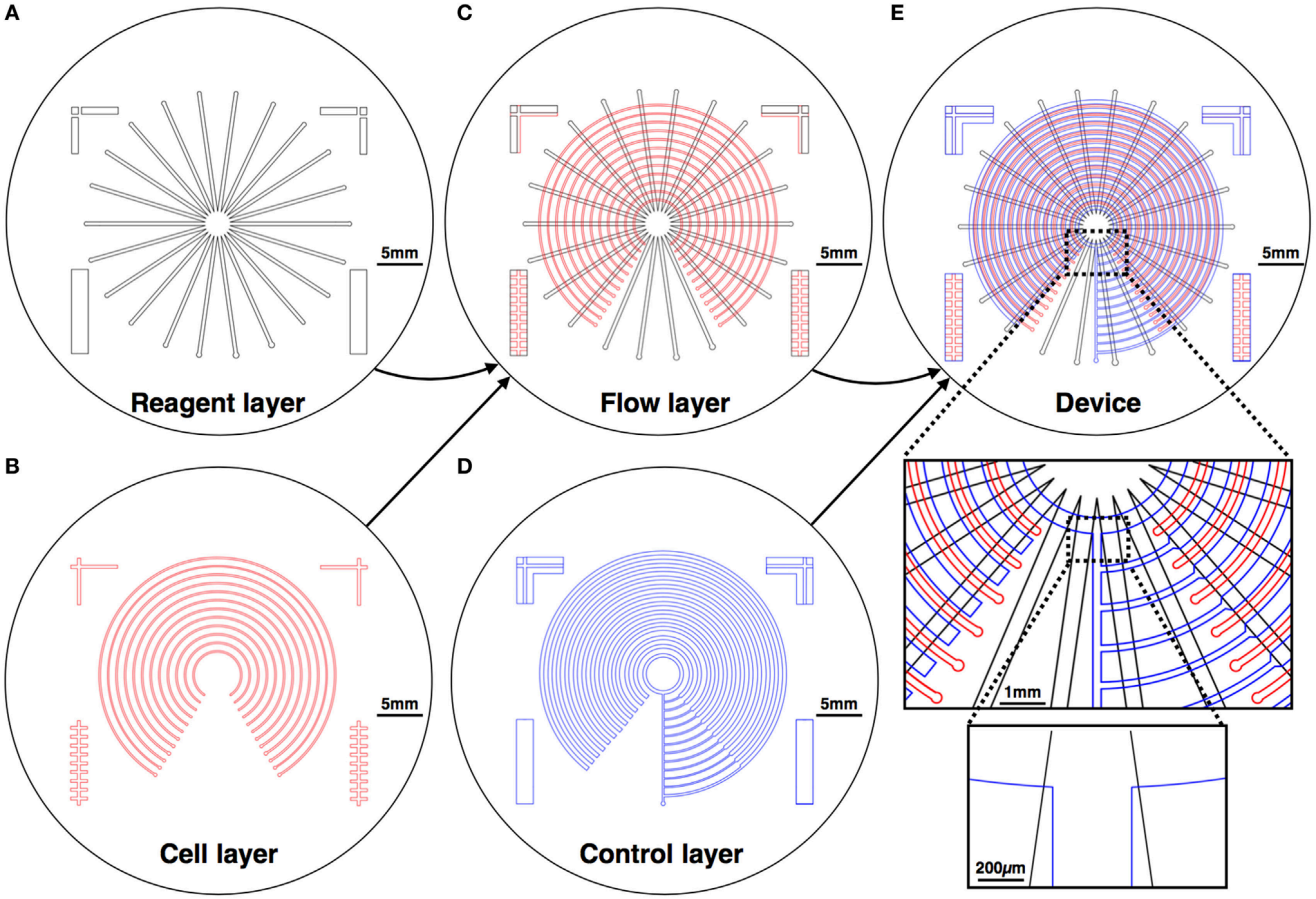
**Mechanical design of the device**. The device is composed of three layers: the reagent layer **(A)** and the cell layer **(B)**, which are aligned on top of each other to create a composite flow layer **(C)**; and a control layer **(D)**, which is fabricated separately and then aligned and bonded on top of the flow layer to create the assembled device **(E)**. Chemical reagents are perfused in the reagent layer, and cells are cultured in the cell layer, where the gradients are formed.

**Figure 2:**
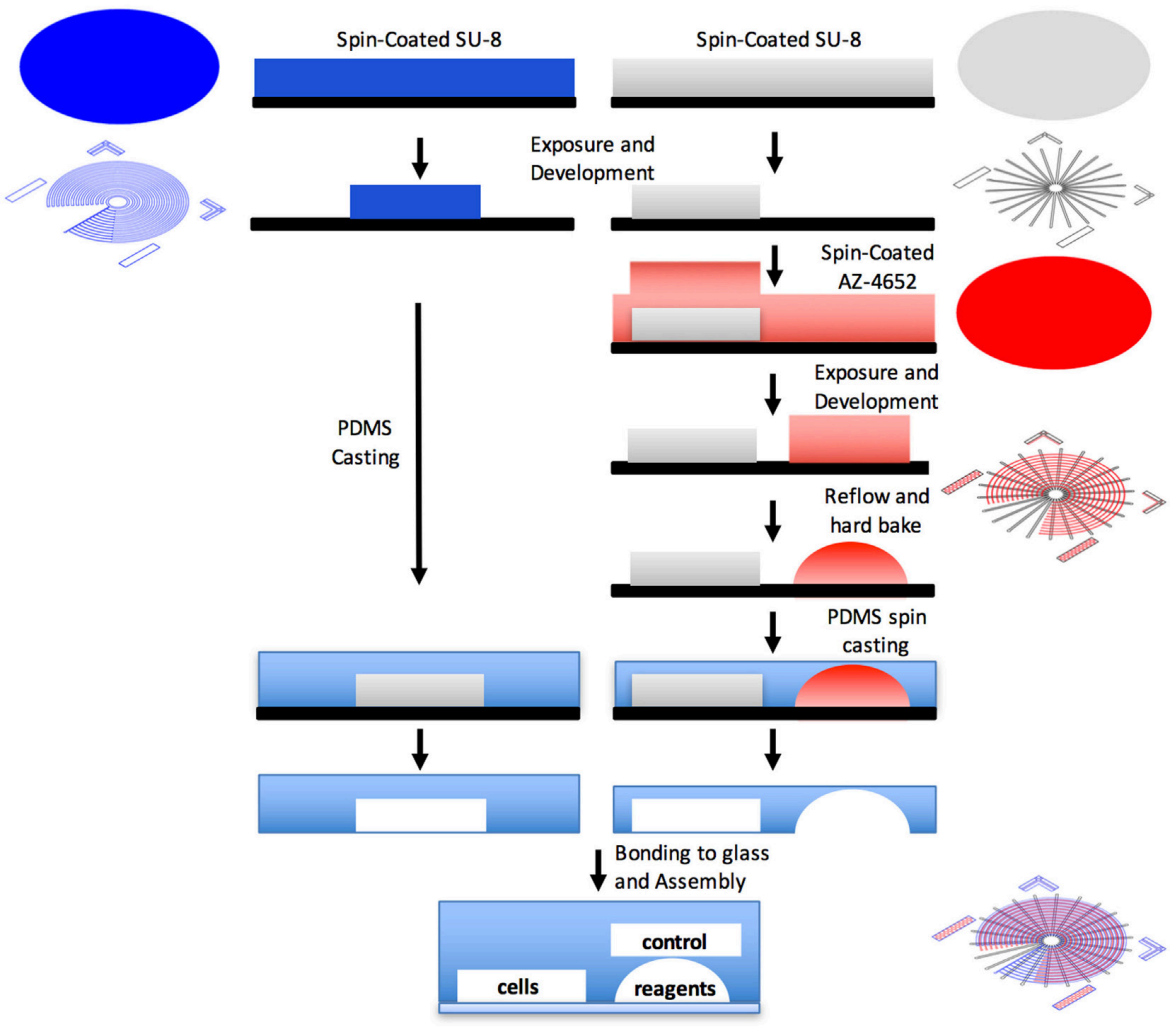
**Schematic of a “push-down” micromechanical valve**. A membrane is formed at the intersections of the control and flow channels, creating a push-down valve.

**Figure 3:**
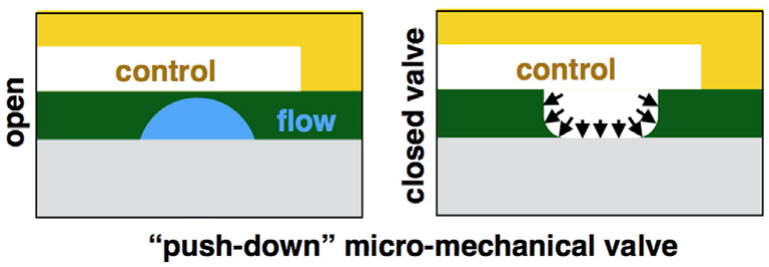
**Illustration of the device fabrication**. First, a reagent layer and a cell layer are created using reflowed positive photoresist and a negative photoresist, respectively. Both patterned on a single silicon wafer. The control layer is molded on a different silicon wafer and fabricated separately. Each layer is separately casted by replica molding of PDMS. The control layer is aligned on top of the flow layer, forming membranes where the control and flow channels intersect orthogonally, creating a push-down valve.

Our design creates varying concentrations of diverse stimulations on an array of different cell lines. The device is formed using three layers: a reagent layer and a cell layer (Figures 1A,B), which are composite layer formed by aligning the cell and reagent layouts on top of each other (Figure 1C), and a control layer (Figure 1D), which is separately fabricated and then aligned and bonded on top of the flow layer to create the assembled device (Figure 1E). The device consists of an array of circular channels, which can be seeded with different cell lines. To be able to seed different cell line in each of the concentric channels, a single pressure actuated microstructured switch was designed. The switch design can seal all intersections between the concentric channels and the flow channels in a single actuation, in a push-down valve formation (Figure 2). In our design, the circular channels are crossed with an array of straight channels alternately, delivering highly concentrated chemicals and buffer to the middle of the device. In this way, an array of diffusive gradients is created. A fabrication schematic of the design is illustrated in Figure 3. As opposed to the circular reagent channels, the cell channels are rectangular, creating homogenous conditions for the seeded cells. We note that a minimal pressure difference of 4–5 Pa between the control and flow channels is essential to gain tight sealing as will be exemplified later. Cross-sectional sizes are 40 µm × 30 µm and 60 µm × 60 µm for cells and reagent channels, respectively. Device size is 40 mm × 50 mm in size (top view). Minimal feature size is 30 µm.

**Figure 4:**
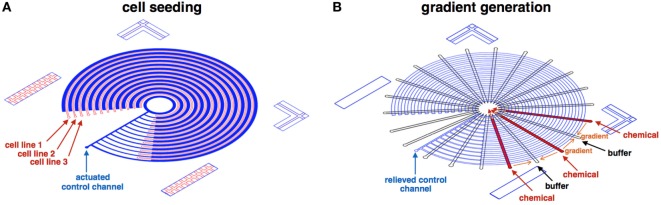
**Illustration of the cell seeding and gradient generation phases**. **(A)** At the cell seeding phase, the control channel (blue) is actuated, separating the concentric cell channels (red) from each other. Cell lines are perfused and seeded to the desired confluency level in each concentric channel. **(B)** At the gradient generation phase, the control layer (blue) is relieved, and highly concentrated chemicals (red)/buffer (clear) is perfused through the reagent channels, generating linear gradients (orange).

Following fabrication, device operation includes three main steps: (1) device preparation, (2) cell seeding (Figure 4A), and (3) gradient generation (Figure 4B). First, the device is coated with 2% Pluronics F68 (P5556; Sigma-Aldrich), which lower particles adherence to the glass and PDMS surfaces (Surface passivation). Next, the control channel is actuated, separating the concentric cell channels from each other. Cell lines are perfused into each concentric channel, seeded to the desired confluency level. Finally, the control layer is relieved, and highly concentrated chemicals/buffer is perfused through the reagent channels, generating linear gradients on-top of the seeded cells.

### Analysis

The diffusion in the concentric channel direction (*x*-direction) can be described with Fick’s law:

(1) Jx=−D∂c∂x

where *J_x_* is flux of particles in the *x*-direction, *D* is the particles’ diffusion coefficient, and *c* is the particles’ concentration. Assuming that concentration varies only along the *x*-direction, maintaining a steady concentration in the flow direction, and that the particles’ concentration in the buffer solution is neglectable, Eq. 1 can be reduced to a linear form:

(2) Jx=Dcl

where *c* is the initial concentration entering the device, and *l* is the location along the *x*-direction. In our design, *x*-axes are stretched on the arcs connecting high concentration points to low concentration points in each concentric channel, where no flow is apparent. Therefore, our design permits the generation of multiple linear gradients along each concentric channel. Note that each gradient is created twice, one in each side of the high concentration point. In our design, some channels are perfused and some are not (no-flow regions). The flow rate in the perfused channels does not have a biological significance, since the liquid is not acting on the cells. However, flow rate does have significance when considering our base assumption for a neglectable concentration differences at the flow direction. Therefore, the desired flow withdrawal rate should be derived from a numerical model that encapsulates the diffusibility and fluid properties of reagent. Intuitively, considering the traveling time of the perfused fluid, the higher the velocity is, the more similar the resulted gradients will be. Since the channels are small, the changes in accuracy are neglectable for most microfluidic applications.

**Figure 5:**
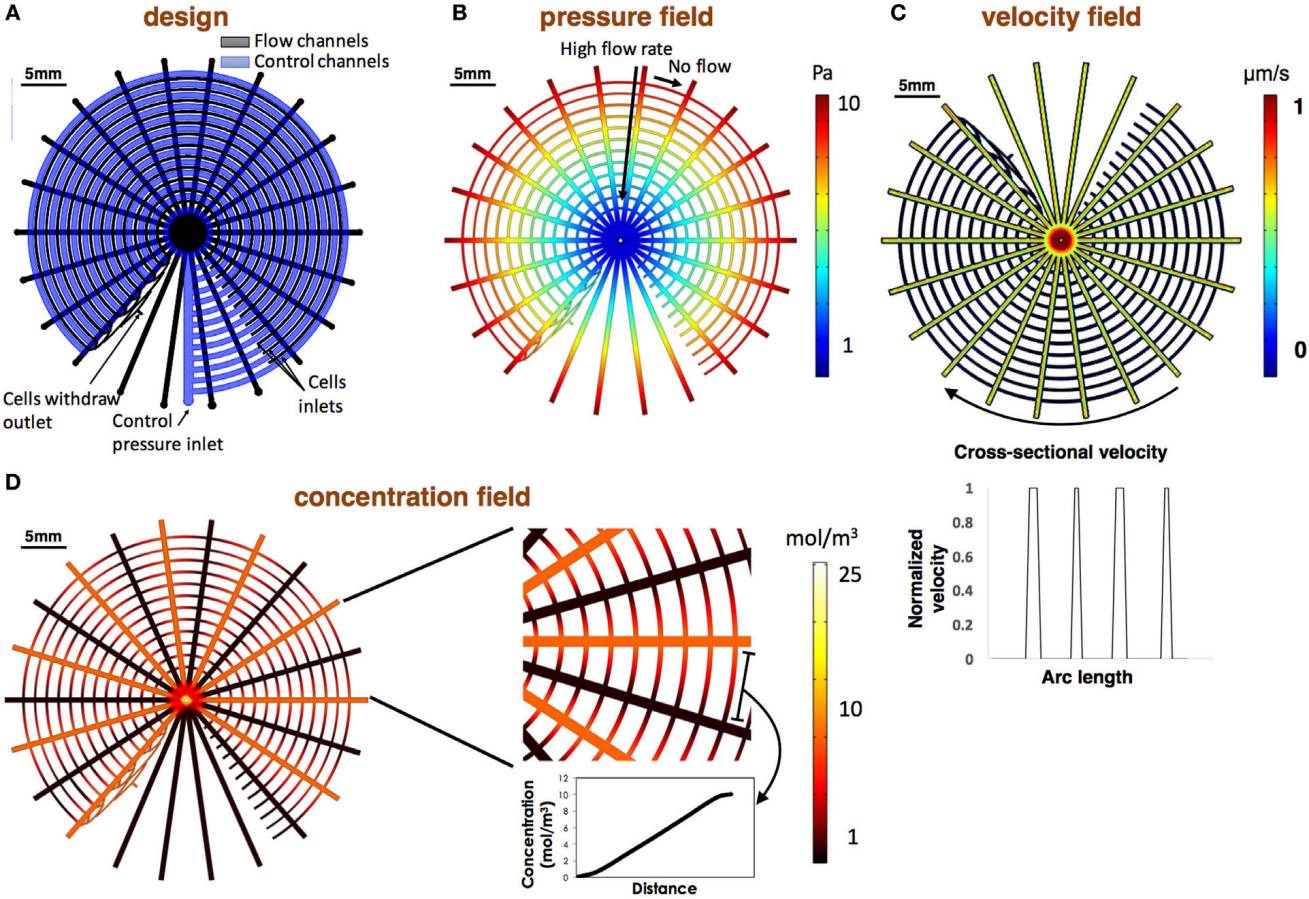
**Design and numerical modeling of the design**. **(A)** CAD of the device. Flow channels are colored in black and control channels in blue. Valves are located at the intersection points of the control and flow layer. **(B,C)** Numerically evaluated pressure and velocity fields. Simulations show that the flow pressure is equilibrated in each of the concentric channels resulting in high flow rate in the stimulations channels and no flow in the concentric channels. Normalized velocity along the concentric channels is shown at the bottom of the section. Velocity was normalized to the maximum calculated flow rate. **(D)** Numerically evaluated concentration map. Results demonstrate the gained linear gradients in each of the concentric channels.

To verify our design and to estimate the gained gradient in every circle for data analysis, we numerically modeled the device in COMSOL Multi-physics. Device schematics are shown in Figure 5A. Results show that the pressure is equilibrated in each of the concentric channels, causing high flow rate in the stimulation channels and static environment in the cell channels (Figures 5B,C). Normalized velocity along the concentric channels is shown in Figure 5C. Results were used to set the desired withdrawal flow rate from the stimuli channels to ensure that the different reagents are not mixed and that the concentration differences at the flow direction is neglectable (<0.01), as was described above. In our experiment, inlet chemical concentration was set to 1 mol/m^3^ with a diffusion coefficient (*D*) of 1.8 × 10^−9^ m^2^/s. Under these conditions, the model predicts that a neglectable concentration differences at the flow direction is gained with an inlet pressure of 1 Pa and higher. Model prediction shows that the gradients along each of the concentric channels are linear as expected (Figure 5D).

### Experimental Validation

**Figure 6:**
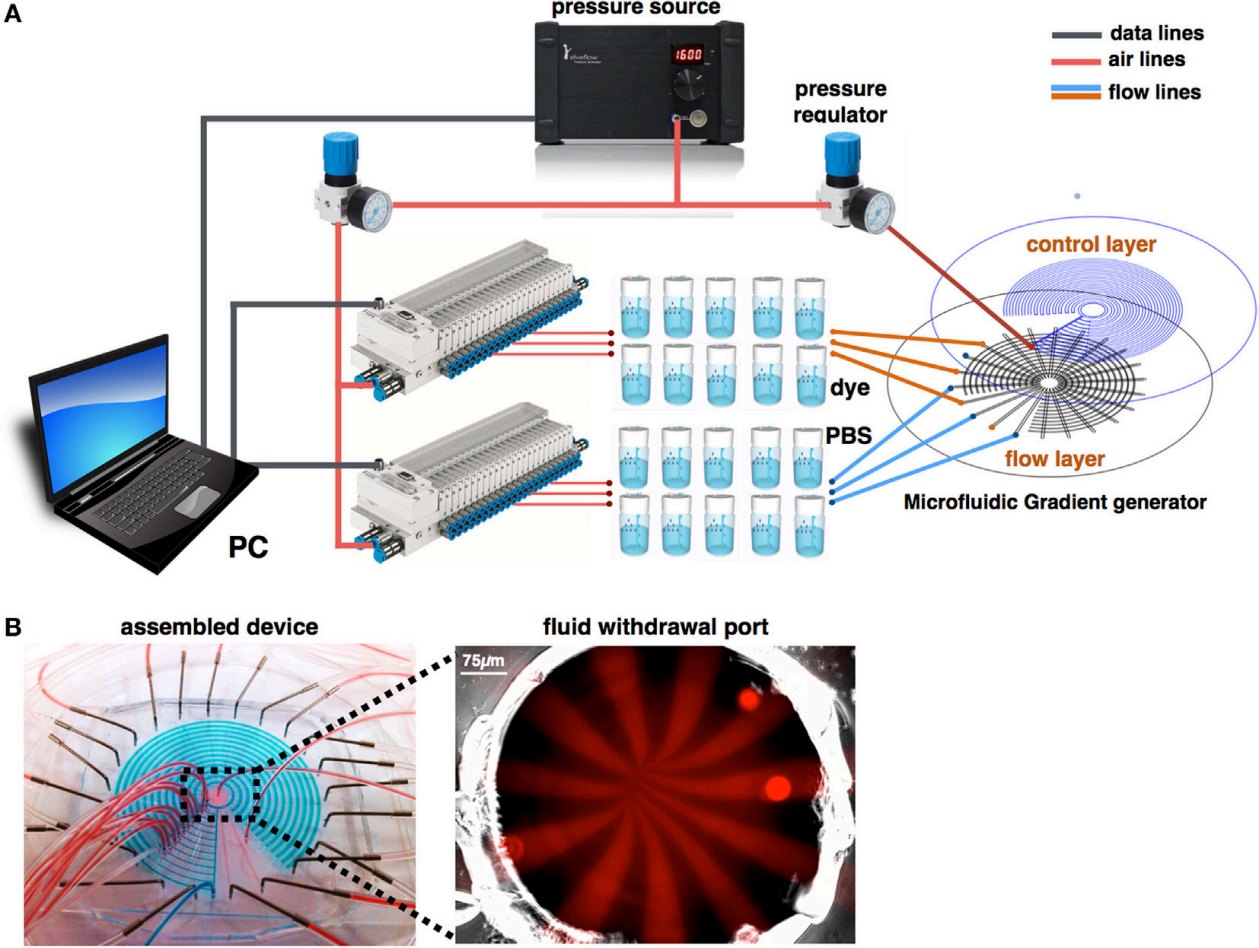
**Experimental design**. **(A)** A pressure source is connected to two pressure regulators that set the switch actuation and flow pressures. Flow pressure is connected to pressurized fluid reservoirs and controlled via two manifolds. **(B)** Fully assembled device. Flow channels are simultaneously perfused with red color dye. Control channels are pressurized with blue color dye. Flow channels are simultaneously perfused with Rhodamine, while the fluid withdrawal port is imaged using fluorescent microscopy. Image shows the perfusion pattern of alternate chemical buffer solutions.

The experimental design is shown in Figures 6A,B. Briefly, two pressure regulators were used to create the pressure differentiation required for controlling the microfluidic switch and to perfuse the chemical stimuli. Fluids were driven through pressurized fluid reservoirs. Since our design consists of 22 inlets that should be perfused simultaneously, the flow pressure regulator outlet was split into two manifolds, in which each of the outlets are digitally controlled. The device was continuously monitored by automated microscopy.

**Figure 7:**
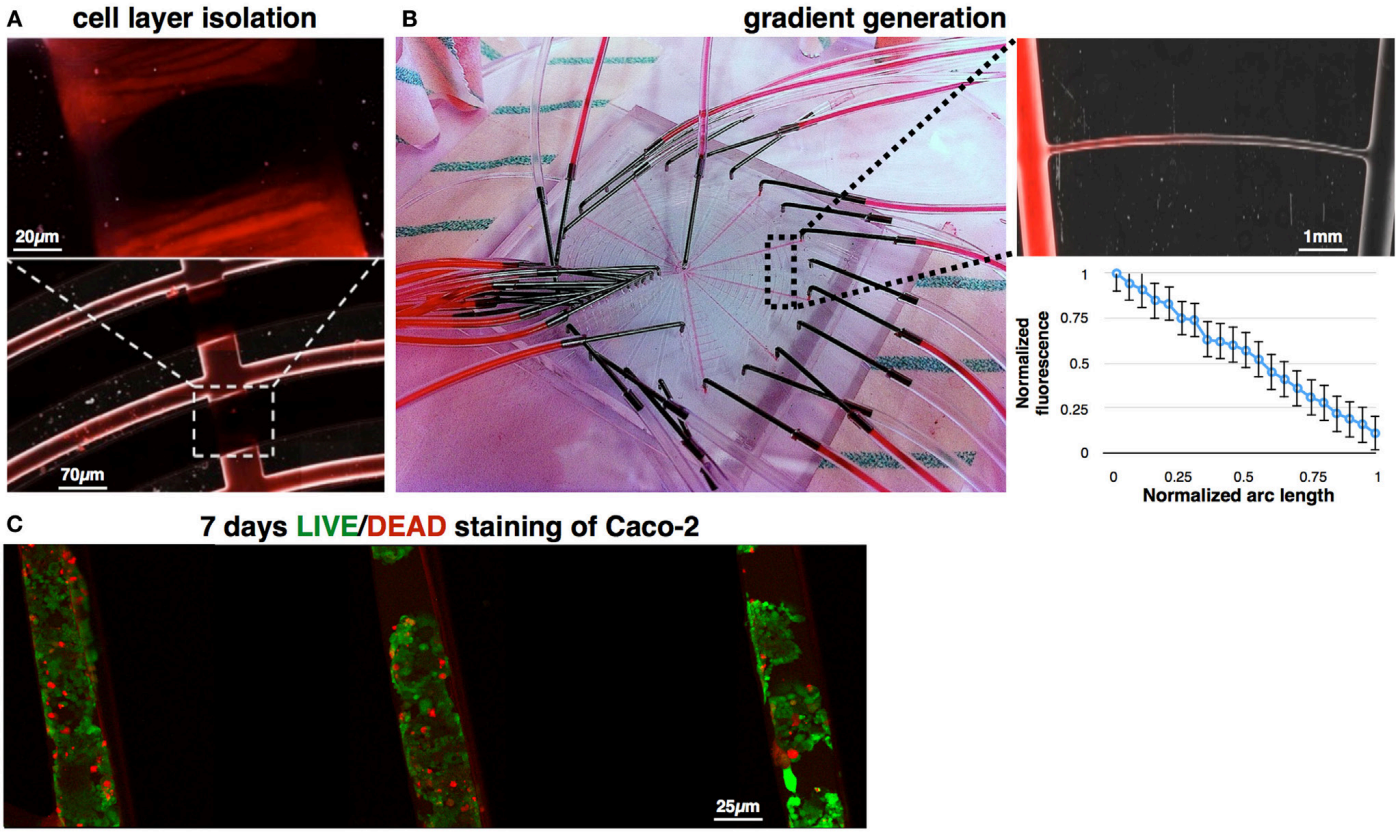
**Demonstration of switch actuation and gradient generation**. **(A)** Concentric channels were perfused with Rhodamine (0.6 Pa), while actuating the control channel (6.6 Pa). Fluorescent imaging show perfect sealing of the flow channels, allowing separation between all concentric channels during cell seeding. **(B)** Stimulations channels were perfused with alternate Rhodamine-buffer sequence, generating linear gradients of Rhodamine along the concentric channels. Fluorescence imaging demonstrates one of the gained gradients (up). Fluorescence level was quantified across all concentric channels. Length of each arc was normalized, and the fluorescence intensity was measured in fixed locations (bottom). **(C)** LIFE/DEAD staining of Caco-2 cells, which were cultured in the device for 7 days. Image analysis shows viability >85% in day 7.

To verify the switching mechanism, the switch was closed while Rhodamine—a fluorine dye—was introduced to the cells channels in an inlet pressure of 1 Pa and a concentration of 2 × 10^6^ particles/mL. Rhodamine was perfused at velocities ranging from 0.001 to 0.1 m/s. Results show perfect sealing of the 60 μm height channels (Figure 7A), in all the tested velocities, when a pressure difference of 6 Pa was preserved using controllable digital pressure regulators (Ezra et al., 2015). To verify the gained gradient in the device, Rhodamine and a buffer were alternatively introduced to the device’s inlets. Fluorescence imaging revealed dye gradients, which were generated along the concentric channels. Results were quantified using Zeiss ZEN software and show linear gradients, in agreement with our simulations and analysis. Since the generated gradients are similar in every arc, we normalized the arc lengths and measured the generated gradients in fixed locations. Data were averaged as shown in Figure 7B. Experiment was repeated four times and averaged across all generated gradients. Finally, we demonstrate cell culture in the device (Figure 7C). Briefly, Caco-2 cells were perfused, seeded, and maintained in the device for 7 days with daily media change. In day 7, cells viability was quantified using a LIVE/DEAD fluorescence assay. Image analysis shows viability >85%.

In this proposed design, 12 different cell lines can be cultured and exposed to 16 chemical gradients (since each gradient is created twice, 8 unique gradients are generated), achieving an array of 96 gradient-based experiments. In our experimental setup, phase and fluorescence images were captured at preprogrammed locations, in a discretized manner, each gradient was monitored in 8 non-overlapping regions, resulting in 768 measurements per scan. Time for each measurement and location shift is ~4 s, resulting in a scan time of about an hour. In our experimental setup, we performed a complete scan every 90 min producing 18,432 data points in a 36-h experimental matrix.

## Discussion

Diffusible signaling molecules play important roles in various *in vitro*/*in vivo* processes. Hence, great efforts were made to obtain stabilized chemical gradients (Takayama et al., 1999; Kim et al., 2010). Microfluidics technology holds great promise to provide versatile technology to accurately control tempospatial conditions (Weibel and Whitesides, 2006). Various designs were proposed to generate chemical gradients in microfluidics devices. While providing powerful platforms for the study of gradient-induced phenomena such as cellular differentiation and migration processes, current designs are often limited in throughput. Some limit the number of obtained chemical gradients and others the number of cells types’ simultaneously undergoing gradient stimulation. Here, we utilized micromechanical pressure-actuated valve design (Melin and Quake, 2007) to allow easy seeding of different cell populations in a single device in one phase of the experiment and then generating an array of stable gradients in the second phase. Our digitally controlled device can be automated by synchronizing the different phases.

Our system can be utilized for various studies. For example, distinct GFP-reporter cell lines encompassing major nuclear receptors can be seeded in each concentric channel and then exposed in the perpendicular direction to a gradient of multiple soluble agonist or antagonist factors. This creates a 3-D matrix of experiments, where each row defines the dynamic response of multiple nuclear receptors to a gradient of a single stimulus, and each circle defines the dynamic response of a single nuclear receptor to gradients of multiple stimuli. At the core of this technology lies GFP-reporter constructs, which are generated by cloning multiple repeats of a DNA response element, upstream of an inducible CMV minimal promoter (Sørensen et al., 2005). The binding of the active form of the transcription factor to its DNA response element induces the transcription of destabilized copGFP with a half-life of 1 h, allowing the signal to rapidly decline when activity decreases. The generated gradients can be quantified using automated time-lapse fluorescence microscopy, allowing monitored in real time and enabling measurements of numerous data points in any given time frame. Results can be used, for example, to model the connectivity and dynamics of a transcriptional network (Levy et al., 2016). In our hands, the device was utilized to culture huh7, upcytes, and HepG2/C3A cell, for toxicity screening assay (amiodarone and acetaminophen).

We would like to point out two considerations regarding the generated gradients and fabrication. First, as the concentric nature of the channels dictates, the size of cells segments becomes smaller going from outer to inner segments. Therefore, even though each cell segment is exposed to the same linear gradient, the gradient slope is different in every segmental arc. This is in correspondence with the normalized manner with which data are presented in Figure 7B. These should be considered in result analysis. Specifically, fluorescence reading should be matched with the exact location and identity of the cell segments from which it was taken. We would also like to note that since each channel cannot be identical due to the limitation of microfabrication, in some devices, correlating pressure difference with the concentration gradient is not accurate. In our facilities, due to fabrication inaccuracies, 12% of the fabricated devices did not generate all gradients and were therefore not fully operational. In addition, in biological essays, cells or reagent might aggregate and affect diffusion. Mixing low concentration of 2% Pluronics F68 might lower aggregates formation, as well as particles adherence to the glass and PDMS surfaces.

Finally, our computer-controlled device enables rapid generation of multiple chemical gradients in numerous channels, in which different cell lines can be seeded, creating an array of gradient-related experiments in a high-throughput manner.

## Author Contributions

ET and YN designed the device, analyzed the results, and wrote the manuscript; ET numerically evaluated the design; ET and MZ experimentally evaluated the design; ET, AE, and IM fabricated the device.

## Conflict of Interest Statement

The authors declare that the research was conducted in the absence of any commercial or financial relationships that could be construed as a potential conflict of interest.
